# Preliminary report of a Web-based instrument to assess and teach knowledge and clinical thinking to medical student

**DOI:** 10.5116/ijme.52a7.7280

**Published:** 2014-01-04

**Authors:** Gerald H. Stein, Hironobu Tokunaga, Hirotaka Ando, Mikako Obika, Tomoko Miyoshi, Yasuharu Tokuda, Miho Bautista, Hitomi Kataoka, Hidekazu Terasawa

**Affiliations:** 1 Department of Medicine, College of Medicine, University of Florida, Gainesville, Florida, USA; 2 Department of Emergency Medicine, University of Fukui, Faculty of Medical Sciences, Fukui, Japan; 3 Centers for Graduate Medical Education, Okayama, Okayama University Graduate School of Medicine, Okayama, Japan; 4 Department of Medical Education and Primary Care, Okayama University School of Medicine, Okayama, Japan; 5 Mito Medical Center, University of Tsukuba Hospital, Institute of Clinical Medicine, University of Tsukuba, Ibaraki, Japan; 6 Department of Aging and Geriatrics, University of Florida College of Medicine, Gainesville, Florida, USA

**Keywords:** Testing and educating medical students, teaching knowledge and clinical thinking, clinical narrative, Web-based learning

## Abstract

**Objectives:**

We report the preliminary development of a unique Web-based instrument for assessing and teaching knowledge and developing clinical thinking called the “Sequential Questions and Answers” (SQA) test. Included in this feasibility report are physicians’ answers to the Sequential Questions and Answers pre- and posttests and their brief questionnaire replies.

**Methods:**

The authors refined the SQA test case scenario for content, ease of modifications of case scenarios, test uploading and answer retrieval. Eleven geographically distant physicians evaluated the SQA test, taking the pretest and posttest within two weeks. These physicians completed a brief questionnaire about the SQA test.

**Results:**

Eleven physicians completed the SQA pre- and posttest; all answers were downloaded for analysis. They reported the ease of website login and navigating within the test module together with many helpful suggestions. Their average posttest score gain was 53% (p=0.012).

**Conclusions:**

We report the successful launch of a unique Web-based instrument referred to as the Sequential Questions and Answers test. This distinctive test combines teaching organization of the clinical narrative into an assessment tool that promotes acquiring medical knowledge and clinical thinking. We successfully demonstrated the feasibility of geographically distant physicians to access the SQA instrument. The physicians’ helpful suggestions will be added to future SQA test versions. Medical schools might explore the integration of this multi-language-capable SQA assessment and teaching instrument into their undergraduate medical curriculum.

## Introduction

The tradition-bound Japanese medical educational system has been challenged to undergo mandated changes.^1^ Recent reforms of the Japanese medical postgraduate training system have included a required two-year postgraduate clinical super rotation and a ‘Match’ system for selection of postgraduate training sites.^2^ However, the basic undergraduate medical university curriculum remains unchanged with fifth and sixth year medical students undertaking 15-17 two to three week clinical rotations with minimal practical clinical skills training.^3^^,^^4^ Although improvements have been reported, a recent survey revealed 83% Japanese postgraduate first year residents felt incompetent in their clinical skills.^5^ Moreover, errors in cognitive thinking, one of core clinical skills, among physicians were linked to the most important cause for medical litigation cases in Japan.^6^ Solutions to improve clinical skill training at Japanese medical universities have included 1) Ministry of Education mandated core curriculum implementation and testing,^2^ 2) US medical schools conducting skill training workshops at both Japanese and American medical universities,^7^ 3) American medical educators leading Problem Based Learning (PBL) workshops in Japan,^4^^,^^8^ and 4) Japanese medical universities appointing Japanese physicians trained abroad in clinical skills to educational leadership positions.^9^^,^^10^ Recently, we reported Japanese medical student-initiated attempts to improve clinical thinking via real time audio-video Internet based seminars via the Worldwide Web [Webinars].^11^^,^^12^ Lacking from this report was an instrument to show changes, if any, from such webinars. However, other examples for PBL-type evaluation of medical students did not accommodate our need.^13,^^14^ For example, Tsukamoto, Ohira et al. recently reported a useful instrument for teaching clinical thinking which required printed cards with case scenarios and direct tutor guidance.^15^ Thus, we developed an Internet-based program, called the Sequential Questions and Answers (SQA) test, for assessing clinical knowledge and teaching clinical thinking. The SQA test can be formatted into all common international languages. This report is a preliminary description of an Internet-based short text answers program, the first such Japanese language program, and its feasibility as assessed by a brief questionnaire. Additionally, we report the pre- and post-SQA test scores of volunteer physicians.

## Methods

### Study design

We developed two SQA clinical scenarios, the pre – and the posttests that consisted of 8 questions each with common patient-focused narrative, with sequential clinical information, followed by a question requiring brief text written answers, with immediate correct answers upon completion of the text answer. The case scenarios were in the usual sequential clinical narrative form of the history of the presenting complaint, physical examination, laboratory and imaging data, and differential diagnosis.The pre-test was a simplified pneumonia narrative of a patient with fever, cough and sputum; this patient’s narrative reported that the chest X-ray had peripheral infiltration only. The posttest pneumonia narrative was similar with the added long history of cigarette smoking, with a peripheral infiltration and hilar mass on the chest X-ray report, and sputum cytology positive for cancerous cells. The SQA tests were drafted in English, translated and re-edited in Japanese and back translated into English for language consistency. All participants were administered only the SQA Japanese version.

### Data collection and analysis

All tests were taken in Japan on the participants’ geographically distant personal computers at times of their choice during a two-week interval, from January to February 2011 inclusive. The SQA text answers were captured from the participants on a program’s spreadsheet for data retrieval and analysis. The participants’ SQA pre and posttest answers to the eight questions were analyzed after translated from Japanese into English. The authors developed a manual master scoring sheet, based on a weighted scoring system totaling 100 points, expressed as percent of correct answers. More weighted points were awarded for the medical history (24 points) and differential diagnosis (42 points). Three authors independently scored the participants’ answers; they completed the scoring without access to the identity of the participants and universities. Differences were resolved by consensus. No statistics were performed on their differences of their scored answers. We did not estimate validity nor reliability for the SQA tests because of the preliminary pilot study design.Lastly, we developed a ten key word computer generated scoring system for the answers. Computer program development was a combined project of a Japanese software company and all the authors of this report (Imagine Labo, Fukui and Tokyo, Japan).

### Questionnaire

We developed a questionnaire to find errors, problems and suggested improvements for future revisions of the pilot SQA program. The questionnaire consisted of two two-point item questions (yes or no), two five-point Likert item questions, and five free text comment questions about the SQA Internet browser and the content of the test modules. The Japanese authors translated the questionnaire from English after several revisions. We did not estimate validity nor reliability for the questionnaire for this preliminary pilot project.

### Participants

We recruited 12 Japanese volunteer general medicine residents and clinical staff to undertake the pre- and post-practice and test modules. We limited the sample size of the study because this was a restricted pilot study. Of the nine residents, five were first year postgraduates, one was a second year postgraduate, and three were third year postgraduates; two were staff physicians. The 12 examinees represented three geographically distant Japanese medical university hospitals (Fukui, Okayama and Tsukuba). The examinees received no verbal instructs and were not informed about the test topics. In addition to the pre- and posttest modules, the examinees were asked to complete a posttest paper questionnaire about their experiences with the SQA.

### Technical aspects

The SQA Internet program consisted of three parts: 1) an administrative module for uploading the PBL case scenario with questions and answers, and retrieving the examinee’s answers; 2) a practice module for examinees to become familiar with the case navigation and answer entry; and 3) the pre- and posttest test modules. The SQA test was accessed via any universal Internet browser using the dedicated ‘Web’ Uniform Resource Locator (URL) address. The login for the administrative module for uploading the practice and clinical case test modules required a user identification and password. Similarly, the login for each participant required an individual user identification and password for taking the practice module or the clinical case test module. The identity of the each participant and university hospital was encoded for confidentially with a unique login and recorded answers.The following are the paths for participants to use the SQA. Familiarity with the test site was experienced initially with the practice module that was made available at the time of the test modules. After logging into a universal secure Internet browser, the participant was guided to the practice module that contained instructions for entering the answers and a series of simple questions, such as ‘name the four seasons’ and ‘what are the primary colors?’ The first window of the first question showed the question and a space to write the text answer. After the participant wrote the answer and clicked the ‘Save’ button, the same window immediately elongated with the original question, its correct answer and the participant’s answer. This was the instant feedback loop of question and answer. No sequential question relationships were programmed in this practice module. Two additional question windows, totaling three unrelated practice questions completed the practice module.In both the pre- and post-SQA test modules a brief review of instructions appeared after the login together with the patient’s brief clinical scenario consisting of the chief complaint and a short history of the presenting illness. The same window showed the first question related to the presenting illness, such as “What additional questions do you ask this patent?’ The participant wrote the brief text answers in a box within the same window that contained the patient's presenting illness information and the question. The participant saved the answer by pressing the ‘Save’ button. Immediately, the same window elongated to contain the question, its correct answers in red and the examinee’s saved answers.Pressing the 'Next' button after reviewing the details of the question and the correct and participant’s answers, the program advanced to the second question. Now the participant could view both the prior completed window of the question and answers, and the next sequential window with new clinical data, a new relevant question and a space for the text answer then appeared.As more information was added in the sequential standardized clinical case presentation order of history, physical examination, preliminary differential diagnosis, laboratory, and final differential diagnosis, the participant was presented with questions and typed brief answers, closed the window ('Save') and the correct information then appeared, all in standard sequential clinical narrative. At any time the participant could scroll up or down to see any prior question, the correct answers with additional clinical data in red, and participant’s own answers. The SQA program restricted the examinee’s ability to change any of their answers once the 'Save' button was pressed. Also, a 30-minute countdown clock, showing the total remaining minutes and seconds, appeared in every new question window of the eight questions. The detailed structure of the software program is beyond the scope of this report. However, the software developers were in continuous consultation with the authors resulting in many revisions. The developers assured the authors that the SQA software could accommodate most common languages, such as English, Spanish, French, Chinese, Russian, etc. This was not tested. Statistical analysis was conducted by using chi-square test for difference of proportions (Table 1) and Fisher’s exact test for difference of the numbers between favorable and unfavorable responses (Table 2). STATA version 10 (STATA Corp., College Station, Texas) was used for all statistical analyses.

**Table 1:** Average percent of correct answers for the 11 physician participants. Sequential Question and Answer (SQA) pre-test and post-test two weeks later, based on a weighted score of 100 points for each of the two tests.

Physician participants	Total % correct Pretest answers	Total % correct Posttest answers
1	46	64.5
2	51.5	66
3	31.5	68.5
4	46	48
5	46	68.5
6	60.5	76.5
7	49.5	76.5
8	45.5	63
9	41.5	83
10	37.5	78
11	37.5	63
Average*	44.8	68.7

*53% Increase total points pre to post (p=0.012)

**Table 2:** The 11 physician participants’ questionnaire responses regarding SQA tests

Question	Number of favorable responses	Number of unfavorable responses	p value*
SQA tests improves learning case presentation	11	-	<0.001
SQA tests evaluates clinical thinking	11	-	<0.001
Did you acquire new clinical knowledge	9	2	0.009

*Based on Fisher’s exact test for difference of the numbers between favorable and unfavorable responses

Ethical approval for educational studies and surveys was obtained from the hospital ethics committee of the Mito Kyodo General Hospital. The study adhered to International Ethical Guidelines. The respondents were informed they were not identifiable from the data and that they could withdraw any time without prejudice.

## Results

Twelve participants took the test modules during a two-week interval from January to February 2011, inclusive. All answers were captured and downloaded on spreadsheets containing both the pre- and posttest answers encoded for each examinee and respective universities. Eleven participants completed the pre- and posttest modules. One participant failed to complete the posttest module. Hence, 11 participants’ data were analyzed, including one participant who admitted reading about the pretest subject ‘pneumonia’ before the posttest. The participants’ pretest weighted correct score averaged 44.8 points (44.8%) based on a weighted score of 100, with a range of 31.5% to 60.5%. The posttest weighted correct score, also based on a weighted score of 100, averaged 68.7%, with a range of 48% to 83%, resulting in an average gain of 53.3% with p=0.011 (Table 1). No participant had a correct pretest score equal or greater than 70%. Four participants had a correct answer posttest score of 70% or greater (p=0.027).Eleven participants completed a posttest questionnaire. All reported the website login was easy, navigation within the practice and test modules was easy, and 30 minutes were adequate to answer the questions. All 11 examinees agreed with the comments that 1) ‘SQA tests improved learning case presentation’ and 2) ‘SQA tests evaluated clinical thinking.’ Eight participants commented on the ease of text answer entry. Nine felt they acquired new clinical knowledge (Table 2).

Some of the participants’ free text favorable comments were as follows: the SQA has an advantage over multiple choice tests to teach clinical thinking; the SQA showed the order of the clinical narrative; without multiple choices, participant was forced to think clinically; immediate feedback with correct answers taught clinical knowledge; asking for differential diagnosis twice, once after the clinical history and physical examination, and again asking for differential diagnosis after the laboratory and imaging data, was very good to teach clinical thinking; the SQA improved efficiency of patient understanding; distant learning on participant’s computer was an advantage.Some of the participants’ free text unfavorable comments were: word processing in Japanese, that is, typing text answers was difficult; the posttest questions were too similar to the pretest questions. Specific recommended changes included using larger fonts; color coding of questions, answers and additional information; inadequate time to answer and read the correct answers; the timer increases test taking anxiety; change the countdown timer for each question rather than total time remaining; and add images such as chest X-ray and ECG. These suggestions will be incorporated into future SQA versions.

Because this was a feasibility study, no attempt was made to validate the assessing methods for clinical knowledge or teaching clinical thinking. Although key words where programmed to score the answers to each question, Japanese language difficulties prevented successful use.

## Discussion

We have pioneered the first brief text answered clinical case scenario Internet-based test for Japanese medical students entering clinical studies. The computer program, the unique SQA test, is designed to assess and teach both medical knowledge and clinical thinking. In addition, the student learns the basics of case presentation, including data organization, problem listing, and analysis, called the medical narrative.The SQA test requires the learner to enter short text answers. There are no multiple-choice questions. SQA incorporates immediate feedback that is a test-enhancing feature.^16^ After a visible timed interval, the participant closes the answer window followed by the correct answer window. Its user-friendly design and unique SQA format were well received by the participants. The results showed the participants were able to access the SQA program. All answers were captured and downloaded on a retrievable and easy-to-analyze standard spreadsheet for both the pre- and posttests.Subjective examinees’ comments were generally supportive of the SQA test as an assessment and clinical thinking process, although no exact assessment and clinical thinking data were analyzed. The SQA test administrator could easily change the clinical scenarios for any primary care or clinical specialty patient problem, from simple to complex, in Japanese, English or other common languages. These features are especially useful for international medical educators.We developed the SQA test to fill the void of its many possible imbedded features. After careful medical educational literature review the authors were unable to find any instrument that simulated the clinical narrative, and that required the examinee to think as if he or she was collecting and analyzing the patient’s information in real time as routinely practiced.

### Study limitations

There are several aspects of the methods used in this project that need explanation because the implications of this unique SQA test may be weakened. The SQA was developed primarily for medical students entering their clinical studies. We chose not use students as participants in this limited feasibility project. The authors selected recent postgraduate physicians as participants rather than medical students. We felt the physicians’ clinical experiences and maturity would provide helpful suggestions for SQA test improvements. Also, we were uncertain about the difficulties of the questions. Lastly, with the anticipated project expanded to medical students, we wanted the participants to be completely naïve about the project because the entire undergraduate fifth year medical student classes would participate in the randomization process.The authors had no prior experience designing a clinical based Internet-accessed instrument. We were uncertain if the software program would be fully operational. The sample size of the 12 physician examinees was selected because the authors understood this was a pilot study of the SQA test. We made no attempt to provide data supporting our claim that the SQA test improved clinical thinking. We conceptualized that the weighted scoring for the history question and differential diagnosis questions provided an indirect measure of clinical thinking.^17^ Further studies are needed to support this claim.In addition, we performed no reliability or validation of the SQA test because of the stated limited pilot directed goal. Hence, the SQA test may not be as useful as designed to improve clinical thinking. This also will be considered in future studies. Our questionnaire lacked the more advanced five-point Likert item, weakening the strength of the responses.We did not anticipate the low scores of the physician examinees. Perhaps these physicians were rather casual in their replies since they knew the SQA test was a pilot study and their scores had no direct or indirect consequence. They had to interrupt their busy patient care duties to complete the SQA test. For consideration is the possibility these resident physicians were not skilled in the clinical topics of the SQA test. Alternatively, the power of the currently used SQA test to improve clinical understanding may not have been designed correctly. The relative failure of the participants to attain higher scores should not deter serious medical educators from innovative SQA test improvements to its basic structure. Also we have no data to understand the reasons their posttest scores improved. The practice effect of the repeated similar posttest is the strongest reason for improvement. A larger sample size may enable analysis between questions of knowledge and those of clinical thinking such as differential diagnosis.The problem with computer scoring via keywords needs further development for mass testing. Currently scoring answers requires manual review. In addition, the SQA test needs capability to include radiologic, electrocardiographic and other imaging data. Innovations in Problem Based Learning (PBL) may be added in updated SQA test versions.^18^ We plan to use the SQA test with a Japanese multi-university webinar demonstration project to measure the change after real time clinical tutoring of medical students entering their clinical skills training year.

## Conclusions

Our limited pilot study has demonstrated the feasibility of the deployment of a unique instrument, the SQA test. Although lacking vigorous supporting data, the SQA test has the potential to guide the medical student entering into clinical training through the challenges of understanding the clinical narrative. With the design features of flexibility of case scenarios, the medical educator places the medical student into a simulated clinical drama educating and evaluating the student in the logical order of the data, clinical knowledge acquisition and clinical thinking.We invite medical educators to join us in the quest to resolve the many limitations of our current SQA test, such as validation studies and automated scoring. We are confident the SQA test has the potential to become an internationally important tool to help medical students begin to master the difficult clinical thinking concepts for their patients.

## 

### Conflict of Interest

The authors declare that they have no conflict of interest.
